# What fuels suboptimal care of peripheral intravenous catheter-related infections in hospitals? A qualitative study of decision-making among Spanish nurses

**DOI:** 10.1186/s13756-022-01144-5

**Published:** 2022-08-19

**Authors:** Ian Blanco-Mavillard, Enrique Castro-Sánchez, Gaizka Parra-García, Miguel Ángel Rodríguez-Calero, Miquel Bennasar-Veny, Ismael Fernández-Fernández, Harri Lorente-Neches, Joan de Pedro-Gómez

**Affiliations:** 1 Hospital Manacor, Palma de Mallorca, Spain; 2 University of West London, Brentford, Middlesex, UK; 3 Imperial College London, London, UK; 4 Hospital San Juan de Deu, Palma de Mallorca, Spain; 5 Servei de Salut de Les Illes Balears, Palma de Mallorca, Spain; 6 Universitat de Les Illes Balears (UIB), Palma de Mallorca, Spain; 7 Health Research Institute of the Balearic Islands (IdISBa), Palma de Mallorca, Spain; 8 Health Research Institute (IdISBa), Hospital Son Espases, Palma de Mallorca, Spain

**Keywords:** Clinical decision making, Peripheral venous catheterization, Catheter-related infections

## Abstract

**Background:**

Peripheral intravenous catheters (PIVC) are commonly used in hospital worldwide. However, PIVC are not exempt from complications. Catheter-related bloodstream infections (CRBSI) increase morbidity and mortality rates, and costs for the healthcare organization. PIVC care is shaped by the complex mix of professional and organizational culture, such as knowledge gaps, low perception of impact of PIVCs on patient safety, or lack of hospital guidelines.

**Aim:**

To explore determinants of decision-making about the prevention of PIVC-BSI among nurses in Spanish hospitals.

**Methods:**

We conducted a descriptive qualitative study with semi-structured interviews in three public hospitals, the Balearic Islands Health Care Service in Spain. We considered hospital ward nurses working routinely with inpatients at any of the three hospitals for enrolment in the study. We approached relevant informants to identify suitable participants who recruited other participants through a ‘snowball’ technique. Fourteen inpatient nurses from the hospital took part in this study between September and November 2018. We employed several triangulation strategies to underpin the methodological rigour of our analysis and conducted the member checking, showing the information and codes applied in the recording of the interviews to identify the coherence and any discrepancies of the discourse by participants. We used the COREQ checklist for this study.

**Findings:**

We identified four major themes in the analysis related to determinants of care: The fog of decision-making in PIVC; The taskification of PIVC care; PIVC care is accepted to be suboptimal, yet irrelevant; and chasms between perceived determinants of poor PIVC care and its solutions.

**Conclusion:**

The clinical management of PIVCs appear ambiguous, unclear, and fragmented, with no clear professional responsibility and no nurse leadership, causing a gap in preventing infections. Furthermore, the perception of low risk on PIVC care impact can cause a relevant lack of adherence to the best evidence and patient safety. Implementing facilitation strategies could improve the fidelity of the best available evidence regarding PIVC care and raise awareness among nurses of impact that excellence of care.

**Supplementary Information:**

The online version contains supplementary material available at 10.1186/s13756-022-01144-5.

## Background

Nurses in hospitals worldwide frequently use peripheral intravenous catheters (PIVCs) [1–3]. In common with virtually all other clinical interventions, the use of PIVCs can result in complications and adverse outcomes for patients, with catheter-related bloodstream infections (CRBSI) one of the worst of these adverse events [4]. As seen in PIVC failure [5, 6], healthcare organisations can incur unnecessary expenses and waste resources, fostering dissatisfaction among healthcare professionals and impoverishing the experience of care for patients [7, 8], who are subject to increased hospital length of stays, morbidity and mortality [9].

The need for nurses to optimise the management of peripheral intravenous catheter-related bloodstream infections (PIVC-BSI) has already been extensively documented [10–13]. However, these studies did not provide insights into any institutional mechanisms likely in place to assure patient safety and quality in PIVC care [14]. The determinants of optimal catheter use can exert an influence at different levels: individual (i.e., often reported gaps in knowledge and skills among nurses [15]), social (i.e., collective perceptions shaping the relative importance apportioned to PIVCs and, by extension, their adverse events [16]) and, finally, organisational (i.e., unclear guidelines [17] or as our group has reported, lack of patient involvement in their self-care [18, 19]).

Healthcare workers must appraise and negotiate the effect of such determinants of practice, ideally using existing evidence. Such evidence, operationalized in clinical practice guidelines (CPGs), typically integrates empirical knowledge as free from bias as possible with the preferences of patients [20]. However, implementing and adopting recommendations within CPGs can be protracted [21] due to clinician perceptions [22], the volume and quality of the evidence [23], and even difficulties to integrate the mandates of different CPGs [17]. Specifically, previous findings from our group suggest that the decision-making of nurses was suboptimal regarding the adoption of CPG recommendations for preventing infectious complications and failure related to PIVC, highlighting behavioural and organisational differences between hospital environments and services [19]. Further exploring individual motivations, barriers and facilitators within organisations would contribute towards understanding the contextual elements that underpin decision-making around PIVC care [24, 25]. Therefore, the purpose of this study was to investigate the determinants of suboptimal decision-making among nurses in Spanish hospital wards for the prevention of PIVC-related adverse events including PIVC failure and PIVC-BSI.

## Methods

### Study design and setting

We conducted a qualitative study using semi-structured interviews to elicit perceptions, attitudes and beliefs about individual, team, and structural determinants of suboptimal PIVC management and care. The findings would strengthen the development of interventions and strategies to foster the implementation and diffusion of evidence-based recommendations in the health care service of Balearic Islands (Spain) [26].

We conducted the study in three hospitals. Hospital Manacor and Hospital Comarcal de Inca are state-funded acute care hospitals and serve a population of 150,000 and 130,000 inhabitants. These hospitals have 224 and 165 beds respectively, treating patients from all clinical specialities except cardiac, thoracic, and neurology surgery. The nursing workforce includes 695 and 474 staff, respectively. The 3rd hospital, Hospital Sant Joan de Deu, is a state-funded, long-term care hospital with 197 beds mainly allocated to patients with chronic health problems or palliative needs. All three hospitals benefit from mature infection prevention and control programmes which include typical structures such as surveillance mechanisms and feedback and education activities, as well as institutional leadership and support including dedicated budget and staffing.

### Participant selection and recruitment

Hospital ward nurses working routinely with inpatients at any of the three hospitals were considered for enrolment in the study. We approached key informants to identify suitable participants, who then in turn recruited other participants through a ‘snowball’ technique. We were keen to include participants from a variety of professional backgrounds and career pathways with a view to exploring rich experiences of managing PIVCs.

Participation was voluntary and without monetary compensation. A total of 28 individuals were approached, 19 (68%) of whom agreed in principle to participate, and with 14 participants finally interviewed. Selection, recruitment, and interviews ceased once data saturation was achieved.

### Data collection

All semi-structured, face to face interviews were conducted by three researchers (two nurses and one psychologist). The interview guide used by Castro-Sánchez et al. [16] was adapted to the Spanish context, supplemented by a systematic review of the literature. The semi-structured interview (Additional file 1) was piloted in May 2018 to aid interview procedure and ensure an unambiguous understanding of the questions. The interviews were scheduled to last ~ 45 min and conducted between September–November 2018 in locations and times convenient for participants. Field notes were made during and after the interview.

The interviews were audio-recorded and transcribed *verbatim*, with answers anonymized before the analysis. The transcripts were also returned to participants for comments and clarifications. An initial coding framework was applied to the interviews, which were then once more offered to respondents for validation of salient codes and themes.

### Data analysis

The analysis of the data was carried out in a continuous and iterative manner aided by ATLAS.ti v7 software. In the inductive phase, transcripts were examined searching for units of meaning, and coded. These codes were grouped under broader categories and subcategories. Each transcript was independently coded by two researchers (HL-N and IB-M) who then met to compare their finding. During the deductive phase, data were analysed from the proposed elements of the theoretical framework and literature review.

We employed several triangulation strategies to underpin the methodological rigour of our analysis, following Guba and Lincoln's approach [27]. Regarding methods, we compared the information collected in the recording of the interviews, the codes applied by both researchers, and the review by the participants to identify the coherence and any discrepancies of the discourse (member checking). In terms of data, two members of the research team shared and discussed their findings with each other. Another strategy to improve rigour was the meticulous development and recording of researchers' reflectivity on any methodological decisions made throughout the study, as well as considering their dual status as clinicians and co-investigators [28]. Finally, the responses and initial analysis were discussed with two additional researchers with extensive experience in implementation science and qualitative research. The Consolidated Criteria for Reporting Qualitative Studies (COREQ) checklist was used (Additional file 2).

### Research team and reflexivity

The knowledge about PIVC care and management by the research team was essential to interpret and contextualise the analysis. Three members of research team (two of which had previous experience in evidence implementation and vascular access research, and one in clinical psychology and social research), facilitated and conducted semi-structured interviews at the three participating hospitals. In addition, none of the researchers were linked with the participants, which allowed them to establish a rapport and fostered an open and frank discussion. The principal researcher is a doctoral candidate in a Translational Research in Public Health and High Prevalence Diseases programme.

### Ethical considerations

This study was approved by the appropriate research ethics committees. All participants were informed about the purpose of the study and their implications. We obtained write consent from participants.

## Results

The interviews lasted an average of 35 min, providing rich data regarding the participants' experiences about decisions on PIVC care to prevent the adverse events. Fourteen hospital ward nurses with a range of ages and clinical experience participated in the study. One nurse declined to participate in the study and two did not attend the scheduled interview. No reasons were given for non-participation. Table 1 presents the characteristics of the participants.

**Table 1:** Demographics and professional characteristics of participants

Participant	Gender	Age (years)	Education level	Clinical experience (years)	Ward specialty	Employment time on ward (years)
1	Female	45	BSc	20	Surgical	11
2	Female	23	BSc	1	Long-stay	1
3	Female	34	BSc	12	Traumatology	2
4	Female	41	BSc	6	Oncology	3
5	Female	43	BSc	20	Surgical	9
6	Male	29	BSc	5	Medical	3
7	Female	29	BSc	3	Oncology	3
8	Female	33	BSc, MSc	13	Medical	9
9	Male	44	BSc, MSc	10	Palliative	4
10	Female	32	BSc	11	Medical	9
11	Female	25	BSc, MSc	4	Neurorehabilitation	3
12	Male	30	BSc, MSc	3	Surgical	1
13	Male	29	BSc, MSc	3	Oncology	2
14	Female	34	BSc, MSc	13	Medical	8

*BSc* Bachelor of Science, *MSc* Master of Science

### Themes

Four major themes were identified related to determinants of care and are presented here together with illustrative quotations: (1) The ‘fog’ of decision-making in PIVC; (2) The ‘taskification’ of PIVC care; (3) PIVC care is accepted to be suboptimal, yet irrelevant; and (4) Chasm between perceived determinants of poor PIVC care and its solutions.

**Fig. 1:**
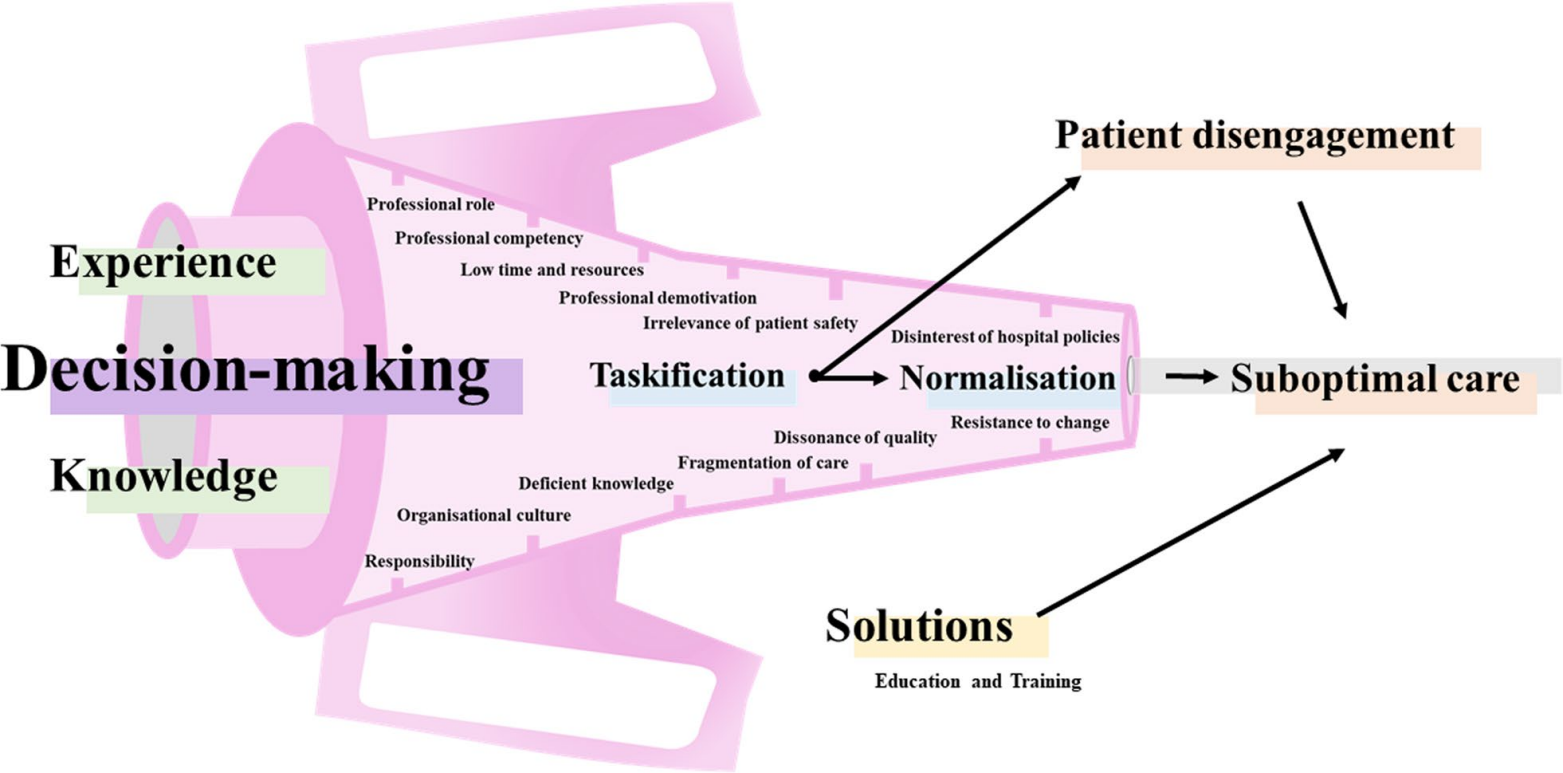
What fuels suboptimal care of peripheral intravenous catheters in Spanish hospitals?

The 15 codes emerged in our study (Additional file 3) indicated that these determinants were connected through PIVC care decision-making. Figure 1 shows what determines optimal care of PIVCs among Spanish nurses.

*(1) The ‘fog’ of decision-making in PIVC* Whilst nurses feel responsible for PIVC care, they do not however see themselves as the responsible decision-maker about PIVC insertion and removal. Such decisions are apportioned to physicians. But such demarcations of responsibility are not however clear cut, with frank ambiguities instead about some clinical decisions. Equally lacking is a jointly agreed upon framework of reference that explicitly allocates roles, professionals, and situations about PIVC care:

I think it's the nurse's responsibility, the decision to insert a catheter, it's the nurse's as well. Well, or the doctor, because sometimes you cannot insert a PIVC, and the doctor decides to put in a central intravenous catheter. Nurse, age 45, surgical ward.

Such ambiguity is reinforced by servile relations between nurses and medical professionals, particularly in cases of urgency, and implicit delegation of tasks. This delegation appears to take place (or is not resented) only when nurses and physicians’ decisions are aligned:

If it is a vital issue for the patient, we (nurses) can insert a vascular access without the authorization of the doctor. If you don't [insert the vascular access], they [doctors] ask you why you haven't inserted it. Yet sometimes when you do it, [insert the vascular access], they (doctors) tell you that you've exceeded your competencies. Nurse, age 41, oncology ward.

*(2) The ‘taskification’ of PIVC care* Decisions about care, maintenance, management, and removal of PIVCs were highly fragmented and conducted as tasks rather than embedded within a nursing care process, resulting in a disjointed and often inefficient experience to reduce potentially infectious complications for patients. This approach to PVC management, which we have named *'taskification',* may be the result of deficient knowledge of professional practices related to PIVC care, or dissonance between the perception of PIVC care offered and care effectively provided, where tasks rather than quality and safety healthcare are prioritised. One participant described this piecemeal approach to care:

*We (nurses) work routinely, performing tasks with automatic habits acquired from the ward. You observe the PIVC, disinfect it, you change the dressing and then you leave*. Nurse, age 45, surgical ward.

*We (nurses) have training. However, we lack an integral view about the management of care. Sometimes, even if you see that the PIVC is in perfect condition, if the patient says that it is hurting, then you should remove the catheter. Some kind of overall awareness is the key to better care.* Nurse, age 43, surgical ward.

This focus on tasks rather than quality is exemplified by the perspectives of participants about the maintenance of PIVCs. Rather than another essential and valuable component of excellent PIVC care, nurses seemed only concerned about carrying it out appropriately to avoid wasting their time reinserting any catheters gone wrong, without a likely reflection on the patient experience or, even worse, implications towards healthcare-associated infections:

*...the interest in maintenance is quite low, what interests you as a nurse is that it takes you time not to do the technique (task) again, but not so much on the subject of infections....* Nurse, age 44, palliative care ward.

*Yes, there are failures, but not everyone fails in the same place. In maintenance people are less careful. People might fail to see a dirty PIVC or get wet because the patient has showered, and they don't change it...* Nurse, age 34, traumatology ward.

Paradoxically, the interest in avoiding any waste of nursing time and resources was neutralised by decisions (or at least, aspirations) to ensure that all patients always had PIVCs, if possible. This blanket approach would appear to fit well with the taskification embedded in the continuum of care, as well as removing engagement for potential disputes with other professionals about the need (or lack of thereof) for PIVCs, an area fraught with uncertainty as previously highlighted:

*I don't want a patient without PIVC, I don't want to, I don't feel safe. I have had some scares and if I can all patients would be carriers of a PIVC. If it were up to me, I would put everyone on a PIVC from the first day to the last day of hospital admission.* Nurse, age 23, long-stay unit.

*(3) PIVC care is accepted to be suboptimal, yet irrelevant* The reduction of PIVC care to an array of tasks surrounded by uncertainty resulting in suboptimal care for patients was acknowledged by the nurses, who tacitly accepted such status quo. Underpinning the inaction was the detachment from hospital policies and best practices, strengthened by their perceived flaws and ambiguity:

*The protocol is outdated and obsolete. I don't think anyone has read it. For example, it recommends routinely changing PIVCs every 96 hours.* Nurse, age 32, medical ward.

These views about clinical practice guidelines as outdated and therefore irrelevant had further unwanted consequences. Often, the disinterest about the policies and the lack of motivation among nurses to adhere and uphold the mandates included in the protocols turned to active resistance against any changes in practice, and even anarchic behaviours:

*Yes, there is a hospital policy, but it's kept in a drawer. No one looks at it, no one teaches about it, but we are expected to know that it is the standard of practice. But people do what they want.* Nurse, age 33, medical ward.

*Perhaps if the hospital establishes a more accessible hospital policy, with clear and precise recommendations for the PIVC care.* Nurse, age 25, neurorehabilitation ward.

In addition, PIVCs were seen as having a low impact on patient safety during the management of intravenous therapy:

*In our ward we have a register of vascular access devices, where the day of insertion and maintenance is recorded. However, I don't do it, I go to the patient and if I have to change the dressing, I do it and that' s it.* Nurse, age 41, oncology ward.

The haphazard approach to patient safety is reflected in some of the behaviours reported by the nurses, who for example recognise that covering the PIVC insertion site can lead to serious complications for patients, yet they frequently engage in that very same practice:

*About the issue of covering the catheter insertion site, many colleagues cover the site when they insert or maintain the catheter. This situation threatens patient safety, but we don't care.* Nurse, age 34, medical ward.

A further dimension of this apparent insensibility to patient safety is the avoidance of patient education within any of the management decisions required during PIVC care:

…It's a relatively simple technique (patient education), which we have very internalized, it seems easy. However, it is difficult to comply with during PIVC care’ Nurse, age 44, palliative care ward.

*(4) Chasms between perceived determinants of poor PIVC care and its solutions* Perhaps unsurprisingly, these failings are justified and normalised as an inevitable consequence of structural deficits in education or training (‘Nurses base their practice on what they learned in university or on day-to-day experience. They do not keep up to date or ignore the evidence…’ Nurse, age 32, geriatric medicine ward), or capital and human resources:

*Lack of time is a resource that hinders us to offer best care. This is nurses’ main complaint* Nurse, age 29, geriatric medicine ward.

*Sometimes you find patients with a true PIVC ‘disaster’, perhaps due to lack of time or workload. Sometimes you don't devote as much time as you would like to PIVC care*. Nurse, age 45, surgical ward.

However, whilst the solutions offered by the participants were aligned with the gaps they suggested existed, with ad hoc training (‘*Above all, we need training to nurses on the ward, even if they just were mini sessions. Ideally, they would be face-to-face or even online courses explaining how to manage and care for PIVCs.’* Nurse, age 32, geriatric medicine ward) or specialised posts with leadership and expertise to mitigate and bring poor PIVC care to the fore (‘*Role models are necessary to provide support, and make explicit the impact of professionals on PIVC, on the importance of optimal care management.’* Nurse, age 41, oncology ward), these interventions would likely be ineffective against the uncertainty and ‘fog’ around decisionmaking, or the shortermism about the PIVC care provided.

## Discussion

Nurses in all clinical settings engage with PIVC care every day, requiring knowledge and skills that include techniques such as catheter insertion, maintenance, and management, together with more social and behavioural skills such as negotiation and communication with other professionals, or patient education. Delving into the motivations and attitudes of nurses was essential to explore the determinants of decision-making about PIVC care (insertion, maintenance, management, and removal) to prevent complications such as PIVC-BSI and their consequences such as sepsis, ICU admission or even death. Our study unearthed how the constellation of decisions associated with PIVC care was highly disjointed, resulting in an erratic pathway for patients and a suboptimal and wasteful process for the healthcare organisation in the struggle against iatrogenic events.

In Spain, the uncertainty and ambiguity reported in other settings [16] is further compounded by a taskification of PIVC care. Nurses outlined their daily PIVC workload along a dated, task-based, nursing model [29]. Future studies further exploring taskification may clarify whether nurses embrace this approach to structuring their nursing work and outputs because it reflects their own views about how nurses should practice, or whether there may be an overarching institutional or professional culture aligned with scientific management theories [30] which promote the division of nursing labour into tasks, which would transfer the intellectual component of care planning and care overseeing away from bedside nurses towards the specialist vascular access nurses as, indeed, reclaimed by some of our participants.

The cognitive impact of such PIVC task-stacking is not known [31], although it is likely to contribute to the increased cognitive work identified by other authors [32]. Further, the taskification may fuel and perpetuate a productivity fallacy whereby engaging in low- rather than high-value work is preferred, as the latter requires an individual and collective effort to assess not only what needs doing, but also what needs to stop [33]. This intellectual effort required by nurses to evaluate and appraise patient care needs, essential but invisible, is unlikely to be captured or documented in task-oriented systems [34].

Besides, nurses that fulfil delegated tasks with no explicit responsibilities and no clinical leadership could provide a sense of satisfaction and comfort during the shift. This stance would hinder nurses from efficiently managing care, as the short-term perspective about the tasks at hand would obstruct a longer-term, more efficient vision which would also be conscious of unnecessary resources used. Such positioning on immediate tasks may also explain the fairly opposed views presented by nurses regarding the quality of their care and the wider PIVC care provided, and the clinical outcomes in this area which group has identified [35].

Healthcare professionals have traditionally perceived vascular access care as poorly related to patient safety, with more frequent adverse events are considered preventable [36, 37]. This perception may justify the irrelevance of PIVC management and encourage the omission of care reported by our participants, individually and collectively, as a rational approach; driven by scarce resources, nurses would opt out of caring for the valueless PIVCs.

Our study does not offer any insight into the views of nurses on patient involvement in shared decision-making related to PIVC. However, participants acknowledged that they did not engage in patient education, arguably the initial requirement for patient implication in care. The consequences of such lack of engagement are not surprising, as seen in other studies reported by our group where we identified that ~ 50% of patients did not know anything about the catheter they carried [19]. These findings are concerning in themselves, but also highlight the missed opportunities to embed patient education about multiple related safety areas such as infection prevention and control, hand hygiene, and vascular catheter care, where patients could have a crucial role [38].

The interventions advocated by the participants to improve their practice focused on mitigating material deficits, but it is unclear how the increased resources would shift the nurses’ view on the impact of PIVCs on patient safety. The proposal for specialist nurses or vascular access specialist teams could improve the quality of the initial insertion and perhaps management of PIVCs [39], but risks, on the other hand, marginalising the interest of general nurses towards PIVCs even more.

Our findings contributed towards understanding the contextual characteristics of various clinical environments as baseline within a quality and safety improvement strategy focused on PIVC care [40]. In that regard, exploring the nursing perceptions related to PIVC care could provide insights into how healthcare professionals construct their decision-making and the core components and contribute to the successful implementation process into clinical practice.

Our study presents some limitations. Our findings are clearly bound to the sociocultural, clinical, and organisational characteristics of the Spanish health and social care system, and the roles explicitly allocated to and implicitly claimed by nursing professionals. We carried out interviews with frontline nurses, and their perceptions of higher-level determinants (i.e., organisational arrangements) may not truly reflect existing institutional policies. Nonetheless, the strength of our study includes that exploring the determinants of optimal PIVC decision-making and management as experienced and constructed by nurses would enable the development of tailored quality improvement interventions [41]. Understanding contextual features is a first required step before effective knowledge transference at different levels [42, 43]. To further understand the interaction between PIVCs policies and stakeholders, we plan to carry out a follow-up study with managers and decisionmakers which will provide contextual insights of meso and macro levels and elicit crucial information on barriers and facilitators.

## Conclusion

In conclusion, our study suggests exploring the determinants of suboptimal decision-making on preventing PIVC-BSI is vital. Uncertainty of responsibility, fragmentation of care coupled with a perception of low risk on the impact and quality of PIVC care can fuel a lack of adherence to recommendations for reducing infectious complications, disempowering patients in their self-care and ultimately harming patient safety.

The implementation of facilitation strategies, including the decision-making about determinants of PIVC care, could improve the adherence to best available evidence, raising awareness among nurses of the impact that care excellence has on patients’ health outcomes.

## Supplementary Information

**Additional file 1**. Interview guide

**Additional file 2**. The Consolidated Criteria for Reporting Qualitative Studies (COREQ) 32-item checklist for manuscript

**Additional file 3**. List of Themes and codes

## Data Availability

The data that support the findings of this study are available on request from the corresponding author. The data are not publicly available due to privacy or ethical restrictions.

## Abbreviations

PIVC: Peripheral intravenous catheter
CRBSI: Catheter-related bloodstream infection
PIVC-BSI: Peripheral intravenous catheter-related bloodstream infection
CPG: Clinical practice guideline
BSc: Bachelor of Science
MSc: Master of Science
