# Supplementary material for: What fuels suboptimal care of peripheral intravenous catheter-related infections in hospitals? A qualitative study of decision-making among Spanish nurses
Source: Antimicrob Resist Infect Control. 2022 Aug 19;11:105. doi: 10.1186/s13756-022-01144-5 (PMC9389778; doi:10.1186/s13756-022-01144-5)
Supplement: Supplementary file 3 — Additional file 3. List of Themes and codes [file 13756_2022_1144_MOESM3_ESM.docx]

SUPPORTING TABLE 2. LIST OF THEMES AND CODES

| **Themes** | **Codes** |
| --- | --- |
| The ‘fog’ of decision-making in PIVC | - Responsibility - Professional competency - Professional role - Organisational culture |
| The ‘taskification’ of PIVC care | - Fragmentation of care - Deficient knowledge - Dissonance between perception care offered vs effectively provided - Low time and resources of nurses - Irrelevance of quality and patient safety - Professional demotivation - Unimportance of patient engagement |
| PIVC care is accepted to be suboptimal, yet irrelevant | - Disinterest of hospital policies - Resistance to change - Low impact of PIVC on patient safety |
| PIVC care gaps reflect behavioural shortcomings, yet solutions proposed only involve education and training | - Education and Training |
